# Self-Rated Health in Middle Age and Risk of Hospitalizations and Death: Recurrent Event Analysis of the ARIC Study

**DOI:** 10.1007/s11606-024-08748-0

**Published:** 2024-04-10

**Authors:** Scott Z. Mu, Caitlin W. Hicks, Natalie R. Daya, Randi E. Foraker, Anna M. Kucharska-Newton, Pamela L. Lutsey, Josef Coresh, Elizabeth Selvin

**Affiliations:** 1 Department of Epidemiology and the Welch Center for Prevention, Epidemiology, and Clinical Research, Johns Hopkins Bloomberg School of Public Health, Baltimore, MD USA; 2 Department of Surgery, Rutgers New Jersey Medical School, Newark, NJ USA; 3 Division of Vascular Surgery and Endovascular Therapy, Department of Surgery, Johns Hopkins University School of Medicine, Baltimore, MD USA; 4 Division of General Medical Sciences, Washington University in St. Louis School of Medicine, St. Louis, MO USA; 5 Department of Epidemiology, the Gillings School of Global Public Health, University of North Carolina at Chapel Hill, Chapel Hill, NC USA; 6 Department of Epidemiology, University of Kentucky, Lexington, KY USA; 7 Division of Epidemiology and Community Health, School of Public Health, University of Minnesota, Minneapolis, MN USA

**Keywords:** self-rated health, hospitalizations, survival analysis, recurrent events, epidemiology

## Abstract

**Background:**

Self-rated health is a simple measure that may identify individuals who are at a higher risk for hospitalization or death.

**Objective:**

To quantify the association between a single measure of self-rated health and future risk of recurrent hospitalizations or death.

**Participants:**

Atherosclerosis Risk in Communities (ARIC) study, a community-based prospective cohort study of middle-aged men and women with follow-up beginning from 1987 to 1989.

**Main Measures:**

We quantified the associations between initial self-rated health with risk of recurrent hospitalizations and of death using a recurrent events survival model that allowed for dependency between the rates of hospitalization and hazards of death, adjusted for demographic and clinical factors.

**Key Results:**

Of the 14,937 ARIC cohort individuals with available self-rated health and covariate information, 34% of individuals reported “excellent” health, 47% “good,” 16% “fair,” and 3% “poor” at study baseline. After a median follow-up of 27.7 years, 1955 (39%), 3569 (51%), 1626 (67%), and 402 (83%) individuals with “excellent,” “good,” “fair,” and “poor” health, respectively, had died. After adjusting for demographic factors and medical history, a less favorable self-rated health status was associated with increased rates of hospitalization and death. As compared to those reporting “excellent” health, adults with “good,” “fair,” and “poor” health had 1.22 (1.07 to 1.40), 2.01 (1.63 to 2.47), and 3.13 (2.39 to 4.09) times the rate of hospitalizations, respectively. The hazards of death also increased with worsening categories of self-rated health, with “good,” “fair,” and “poor” health individuals experiencing 1.30 (1.12 to 1.51), 2.15 (1.71 to 2.69), and 3.40 (2.54 to 4.56) times the hazard of death compared to “excellent,” respectively.

**Conclusions:**

Even after adjusting for demographic and clinical factors, having a less favorable response on a single measure of self-rated health taken in middle age is a potent marker of future hospitalizations and death.

**Supplementary Information:**

The online version contains supplementary material available at 10.1007/s11606-024-08748-0.

## INTRODUCTION

Over the past 40 years, sociological and epidemiological studies have shown that self-rated health is an independent predictor of mortality.^1,2^ Self-rated health is a global summary obtained by asking an individual to categorize their own health as “excellent,” “good,” “fair,” or “poor,” or with a similar scale.^3,4^ Though the original studies focused solely on survival, recent studies of self-rated health have demonstrated its prognostic ability for specific conditions as diverse as osteoarthritis, diabetes, heart failure, and myocardial infarction.^5–10^

In addition to disease-specific endpoints, all-cause hospitalization is an important and more generalized patient-centered endpoint that captures the result of severe disease from any number of causes.^11^ Identifying individuals at higher risk for incident or recurrent hospitalization with a simple interview-based question may help identify people who could benefit from intervention. Previous studies have shown that poor self-rated health is associated with significantly increased risk of hospitalization even after adjusting for social, behavioral, and health-related factors.^12–15^

One challenge of studying the association between self-rated health and hospitalization risk is that sicker individuals also tend to die sooner, meaning that fewer hospitalizations are able to be observed for those with poor health, attenuating this relationship on the population scale. The individual, subject-specific risks could be greater than what population-level parameters suggest, but previous studies of self-rated health have not accounted for these effects on both hospitalization and mortality.

We used data from a large and diverse cohort of middle-aged and older adults in the United States with more than 30 years of follow-up time to evaluate the association between self-rated health and risk of all-cause hospitalization and mortality. We used a joint survival model that accounted for the correlation between hospitalization and death to more accurately estimate the independent effect of self-rated health on both outcomes.

## METHODS

### Study Population

The Atherosclerosis Risk in Communities (ARIC) study is an ongoing prospective cohort of 15,792 adults recruited from four US communities: Forsyth County, North Carolina; Jackson, Mississippi; suburbs of Minneapolis, Minnesota; and Washington County, Maryland.^16^ Participants were between 45 and 64 years old at the time of recruitment into the study, which took place between 1986 and 1989. All participants were asked questions about socioeconomic factors, cardiovascular risk factors, and medical history, as well as self-rated health status.

### Exposure and Outcomes Assessment

Self-rated health was recorded as the response to the question: “Compared to other people your age, would you say that your health is excellent, good, fair, or poor?” Self-rated health was subsequently assessed annually by telephone but only the first response, during the baseline home interview, was used for the analysis.

The outcomes of interest were hospitalizations and deaths due to any cause. Hospital admissions due to any cause were identified during cohort follow-up interviews (annually prior to 2011 and twice-yearly thereafter) and with active community surveillance of hospital discharge records. The hospitalization discharge date was the hospitalization event time, and mortality status and death dates were ascertained using the ARIC cohort follow-up procedures and via the National Death Index.

### Follow-up

For this analysis, participants accrued study time from their individual home interview date until death or administrative censoring. The first home interview date in ARIC was set as the participant recruitment date, and left-truncation was used as necessary to account for differing individual entry dates. Administrative censoring occurred on December 31, 2017, for participants from the Jackson, Mississippi center and December 31, 2019, for other participants. The discrepant censoring dates were because hospitalization records were not yet available from one large hospital in Jackson.

### Analytic Cohort

The analytic cohort comprised participants who had complete baseline information for self-rated health and selected clinical factors: age, sex, body mass index category (calculated from measured height and weight), cigarette use (never, former, or current smoker), and self-reported history of cancer, emphysema or chronic obstructive pulmonary disease, coronary heart disease, myocardial infarction, heart failure, hypertension, or diabetes.^17^ Black individuals from Mississippi and Forsyth County were included, as were White individuals from Forsyth County, Minneapolis suburbs, and Washington County because the sample sizes for other field-center/race groups were too small for stable statistical estimates. These five field-center/race groups were used for adjustment in the final model. Hospitalization events with a discharge date prior to the home interview or after the censoring date were excluded.

### Statistical Analyses

Hospitalization rates were modeled simultaneously along with hazards of death using a semi-parametric joint model that incorporated a frailty term in order to account for the correlation between the recurrent (hospitalization) and terminal (death) events.^18,19^ The recurrent event data was used to estimate a latent participant-specific frailty, which was then used to jointly estimate parameters from a proportional intensity model for the rate of hospitalizations and a proportional hazards model for the hazard of death.^18,20^ This approach relaxes the independent censoring assumption of the standard Cox and Andersen-Gill models and assumes that the estimated frailty has the same multiplicative effect on both the rate of hospitalization and the hazard of death.

Confounders were added as linear adjustment terms in the model. The main parameters of interest were whether the hazard of death or rate of hospitalization differed between individuals of “good,” “fair,” or “poor” health, as compared to those with “excellent” health. We used a non-parametric bootstrap procedure using the subject as the sampling unit to generate standard errors. Significance testing was conducted at an alpha level of 0.05. We report our results as rate ratios and hazard ratios with 95% confidence intervals.

We conducted sensitivity analyses with statistical models that did not account for the potential associations between hospitalization rates and death, using a Cox proportional hazards model to analyze the association between self-rated health and mortality, ignoring hospitalization information, and separately, the Andersen-Gill model to analyze the association between self-rated health and recurrent hospitalization rates, with death treated as an independent censoring mechanism. All analyses were performed in the R language and environment for statistical computing (Version 4.22), using the reReg package for recurrent event modeling.^20,21^

## RESULTS

**Figure 1:**
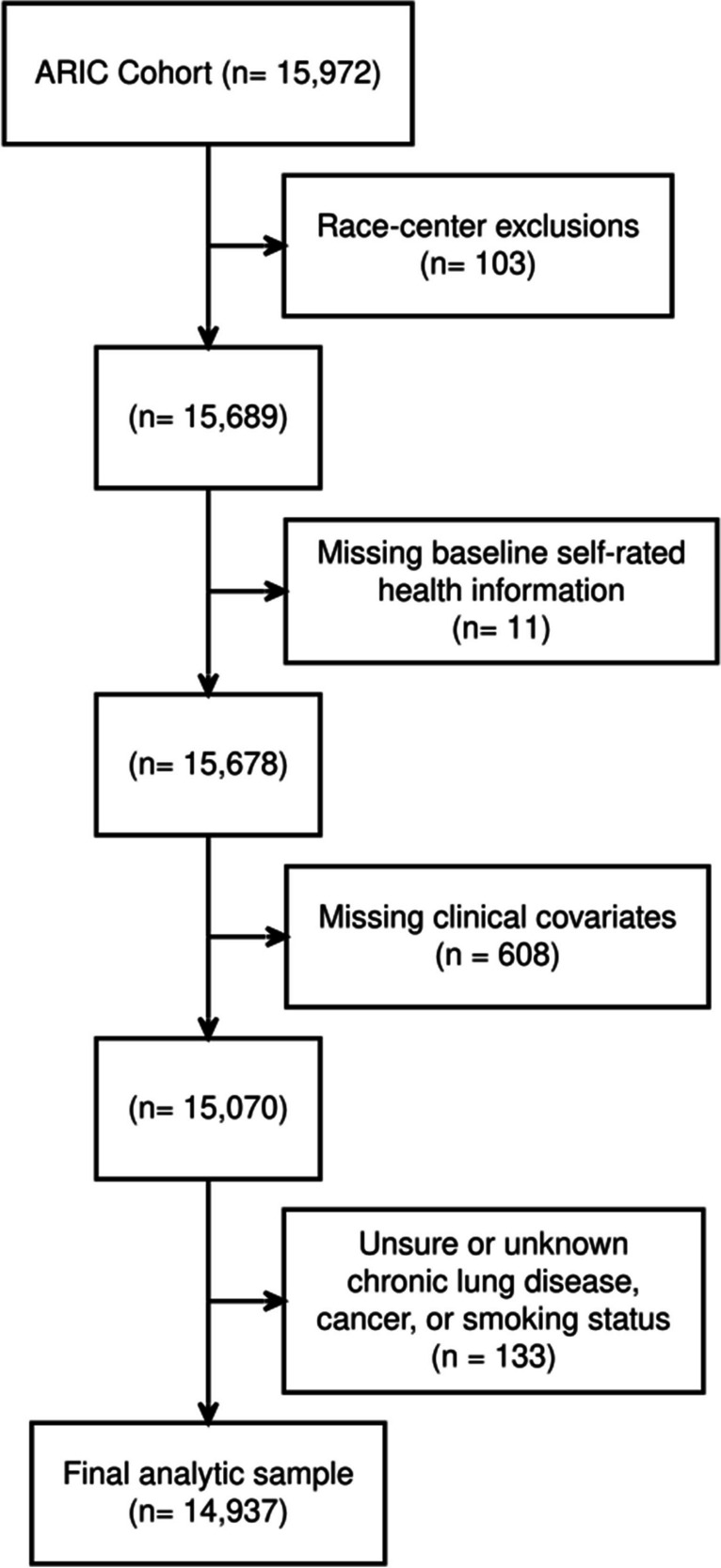
Flow diagram. The inclusion criteria are shown, along with the number of individuals who were excluded. The main analysis was a complete case analysis, including only the individuals belonging to the pre-specified five field-center/race groups with complete demographic and selected clinical factors.

A total of 14,937 ARIC cohort individuals had complete baseline self-rated health and covariate data (Fig. 1). At baseline, participants most commonly reported that, compared to others their age, they had “good” baseline health (47%), followed by “excellent” (34%), “fair” (16%), and “poor” (3%) health. Compared to individuals with “excellent” self-rated health at baseline, individuals with “poor” self-rated health were more likely to be older (57.0 years vs. 53.0 years), obese (44% vs. 18%), and currently smoking (39% vs. 21%) and have a history of cancer (9.5% vs. 4.4%), emphysema/COPD (18% vs. 2.3%), coronary heart disease (21% vs. 1.6%), myocardial infarction (19% vs. 1.3%), heart failure (25% vs. 1.2%), hypertension (67% vs. 19%), or diabetes (39% vs. 4.6%) (Table 1). Those with “good” or “fair” self-rated health had demographic and clinical characteristics that were intermediate, between those of “excellent” and “poor” health.

**Table 1:** Baseline Characteristics

	Excellent, *N* = 5026	Good, *N* = 7002	Fair, *N* = 2426	Poor, *N* = 483
Age	53.0 (49.0, 58.0)	54.0 (49.0, 59.0)	55.0 (50.0, 60.0)	57.0 (52.0, 61.0)
Center (race)				
Forsyth County (Black)	102 (2.0%)	214 (3.1%)	124 (5.1%)	18 (3.7%)
Forsyth County (White)	1358 (27%)	1572 (22%)	384 (16%)	70 (14%)
Jackson, Mississippi (Black)	584 (12%)	1529 (22%)	1097 (45%)	269 (56%)
Minneapolis, Minnesota (White)	1700 (34%)	1843 (26%)	280 (12%)	44 (9.1%)
Washington County (White)	1282 (26%)	1844 (26%)	541 (22%)	82 (17%)
Female	2712 (54%)	3892 (56%)	1352 (56%)	278 (58%)
BMI category				
Normal weight	2089 (42%)	2127 (30%)	508 (21%)	108 (22%)
Underweight	33 (0.7%)	61 (0.9%)	26 (1.1%)	14 (2.9%)
Overweight	2008 (40%)	2843 (41%)	869 (36%)	147 (30%)
Obese	896 (18%)	1971 (28%)	1023 (42%)	214 (44%)
Cigarette use				
Never smoker	2236 (44%)	2882 (41%)	938 (39%)	162 (34%)
Current smoker	1066 (21%)	1866 (27%)	769 (32%)	186 (39%)
Former smoker	1724 (34%)	2254 (32%)	719 (30%)	135 (28%)
History of cancer	220 (4.4%)	392 (5.6%)	168 (6.9%)	46 (9.5%)
History of emphysema/COPD	116 (2.3%)	335 (4.8%)	252 (10%)	89 (18%)
History of coronary heart disease	81 (1.6%)	276 (3.9%)	262 (11%)	103 (21%)
History of myocardial infarction	67 (1.3%)	228 (3.3%)	230 (9.5%)	92 (19%)
History of heart failure	58 (1.2%)	246 (3.5%)	270 (11%)	123 (25%)
History of hypertension	940 (19%)	2566 (37%)	1332 (55%)	325 (67%)
History of diabetes	233 (4.6%)	718 (10%)	636 (26%)	189 (39%)

The baseline characteristics of the analytic cohort are displayed, grouped by initial home interview self-rated health. The median age is displayed with the interquartile range indicated in parentheses. For the other characteristics, the counts of individuals with each characteristic are shown, and the proportion of individuals of that self-rated health category with that characteristic is in parentheses

**Figure 2:**
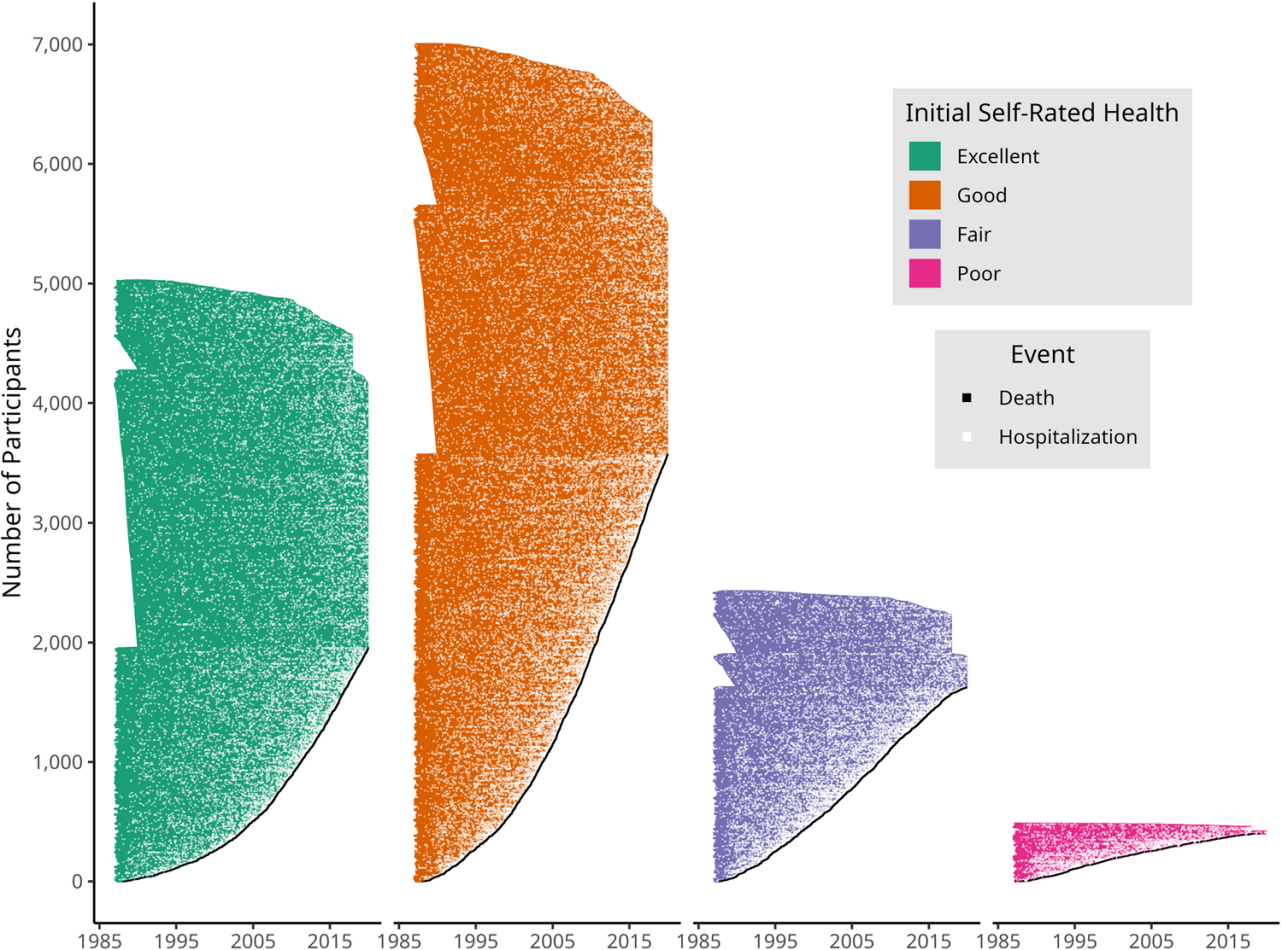
Hospitalization and death events. The hospitalizations and deaths that occurred over time in the analytic cohort are shown. Each thin horizontal shaded bar represents an individual’s observed survival time in the study. Each bar begins at the participant’s entry into the study (home interview) and ends when the participant is deceased or censored. The white points each represent a hospitalization and the black points represent individual participants’ deaths.

The median follow-up time was 30.1 years for those with “excellent” health at baseline, 27.8 years for those reporting “good” health, 22.1 years for “fair,” and 14.7 years for “poor” health. By the end of the study period, 39% of those with “excellent” baseline self-rated health had died, as compared to 51%, 67%, and 83% for “good,” “fair,” and “poor” respectively. A total of 67,295 hospitalizations occurred, 17,866 in those with “excellent” health (27%), and 31,040 (47%), 14,160 (21%), and 3544 (5%) in those with “good,” “fair,” and “poor” health, respectively (Table 2). The hospitalizations and deaths over time are displayed in Fig. 2, grouped by initial self-rated health.

**Table 2:** Unadjusted Event Counts and Follow-up Duration

Initial self-rated health	Number of individuals	Deceased during follow-up	Hospitalization events	Median years of follow-up	Mean years of follow-up
Excellent	5026 (33.6%)	1955 (38.9%)	17,866 (26.8%)	30.1	26.3
Good	7002 (46.9%)	3569 (51.0%)	31,040 (46.6%)	27.8	24.2
Fair	2426 (16.2%)	1626 (67.0%)	14,160 (21.3%)	22.1	20.3
Poor	483 (3.2%)	402 (83.2%)	3544 (5.3%)	14.7	15.5
All	14,937 (100.0%)	7552 (50.6%)	66,610 (100.0%)	27.7	24.0

The total number of individuals belonging to each initial self-rated health category is shown, with corresponding counts of deaths and hospitalization events accrued until censoring. The percentages within parentheses for the “Deceased” column refer to the proportion of individuals of that self-rated health category who died during the study period. The percentages within parentheses for the “Hospitalization events” column refer to the proportion of all hospitalization events that were observed for individuals with a certain self-rated health category

**Figure 3:**
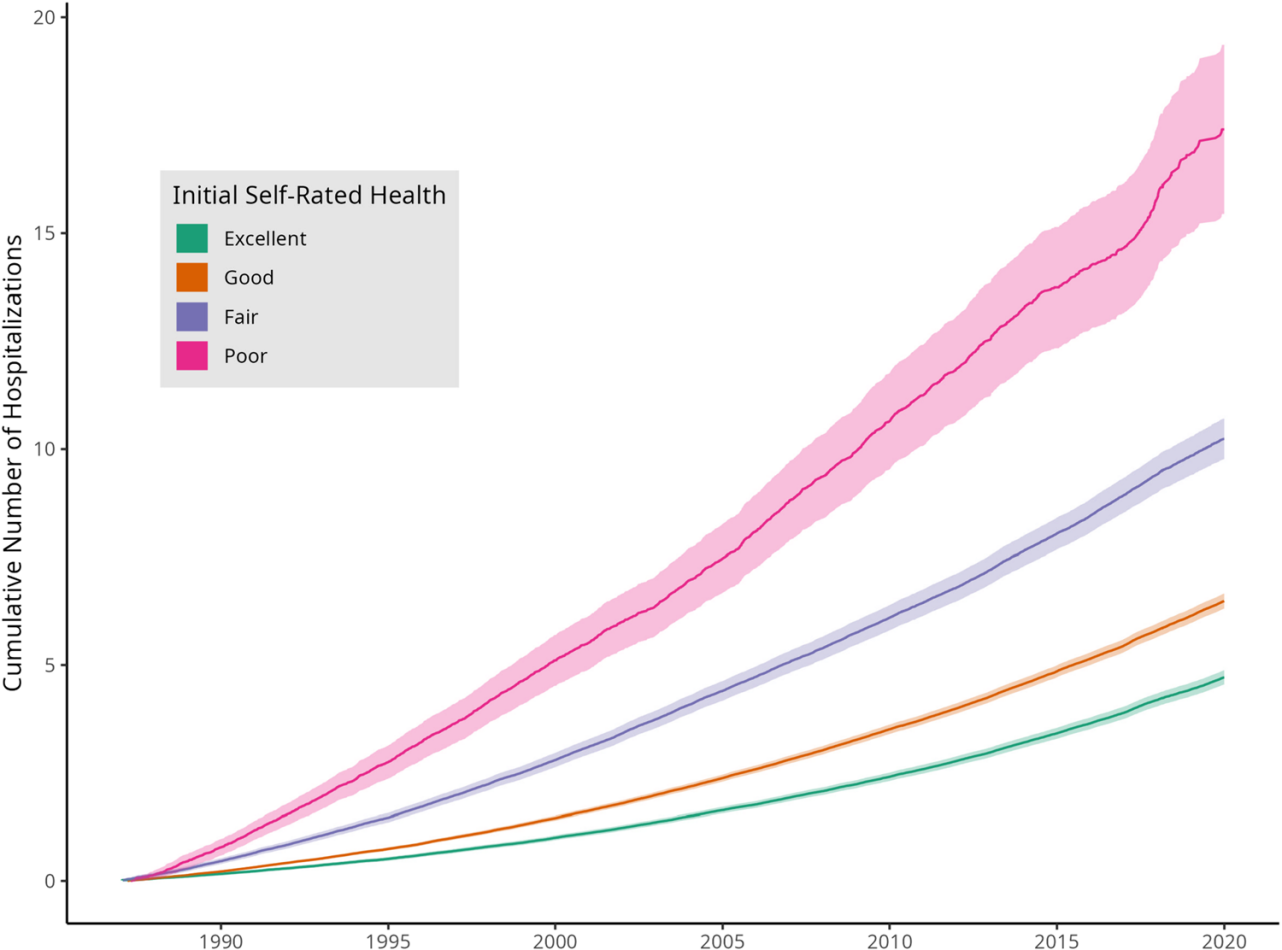
Mean cumulative number of hospitalizations. The Nelson-Aalen estimates of the mean cumulative number of hospitalization events accrued over the entire duration of follow-up are shown, stratified by baseline self-rated health status. This non-parametric estimate takes into account the shrinking risk set over time as individuals die or become censored, but does not take into account the potential violation of the independent censoring assumption that time to death is non-informative with respect to number of hospitalizations.

The Nelson-Aalen estimates of the expected number of hospitalizations accrued by an individual who did not experience death or administrative censoring, stratified by baseline self-rated health category, are shown in Fig. 3 and Appendix Table 1. Those individuals reporting “poor” health had on average 16.9 (15.0 to 18.7) hospitalizations by the end of follow-up, as opposed to those with “good” health, who experienced on average 9.8 (9.4 to 10.3) hospitalizations. Those with “fair” and “excellent” health experienced an average of 6.1 (6.0 to 6.3) and 4.4 (4.3 to 4.6) hospitalizations by the end of follow-up, respectively.

After adjusting for demographic and baseline medical history factors and accounting for non-independent censoring, those with “poor” self-rated health had a 3.13 (2.39 to 4.09) times higher rate of hospitalization compared to those with “excellent” baseline self-rated health (Table 3). Individuals with “fair” health had a relative rate of 2.01 (1.63 to 2.47) and individuals with “good” health had a relative rate of 1.22 (1.07 to 1.40). Similarly, the hazard of death for those with “poor” self-rated health was 3.40 (2.54 to 4.56) times that of “excellent” self-rated health individuals. Those with “fair” health had a hazard ratio of 2.15 (1.71 to 2.69) and those with “good” health had a hazard ratio of 1.30 (1.12 to 1.51). The full model results for all covariates are displayed in Appendix Table 2.

**Table 3:** Risk of Hospitalization and Death by Self-Rated Health Category

		Hospitalizations		Deaths	
Initial self-rated health		Rate ratio (95% CI)	*p*-value	Hazard ratio (95% CI)	*p*-value
Model 1 (unadjusted)					
	Excellent	Reference	—	Reference	—
	Good	1.51 (1.31 to 1.73)	< 0.001	1.67 (1.43 to 1.95)	< 0.001
	Fair	3.42 (2.86 to 4.10)	< 0.001	4.19 (3.43 to 5.12)	< 0.001
	Poor	7.12 (5.65 to 8.98)	< 0.001	9.55 (7.43 to 12.28)	< 0.001
Model 2 (adjusted for demographic factors)					
	Excellent	Reference	—	Reference	—
	Good	1.42 (1.22 to 1.65)	< 0.001	1.55 (1.31 to 1.84)	< 0.001
	Fair	3.01 (2.51 to 3.62)	< 0.001	3.50 (2.87 to 4.27)	< 0.001
	Poor	6.06 (4.71 to 7.78)	< 0.001	7.51 (5.61 to 10.06)	< 0.001
Model 3 (adjusted for demographic and clinical factors)					
	Excellent	Reference	—	Reference	—
	Good	1.22 (1.07 to 1.40)	0.003	1.30 (1.12 to 1.51)	0.001
	Fair	2.01 (1.63 to 2.47)	< 0.001	2.15 (1.71 to 2.69)	< 0.001
	Poor	3.13 (2.39 to 4.09)	< 0.001	3.40 (2.54 to 4.56)	< 0.001

Model 1 shows the unadjusted rate ratios for hospitalization and hazard ratios for death, using a joint frailty model that assumes a person-specific multiplicative frailty that accounts for the correlation between these two outcomes. The estimates in Model 2 were adjusted for age, sex, and race-center category. The estimates in Model 3 were adjusted for age, sex, and race-center category, as well as body mass index category, cigarette use, and history of cancer, emphysema or chronic obstructive pulmonary disease, coronary heart disease, myocardial infarction, heart failure, hypertension, or diabetes

The hazard and rate ratios for self-rated health categories and death or hospitalization events obtained from the joint frailty model were more extreme than the estimates obtained from standard Cox proportional hazards regression and the Andersen-Gill model, but all modeling approaches demonstrated statistically significant increases in the risk of death or hospitalization with worsening categories of baseline self-rated health (Appendix Table 3 and Appendix Table 4).

## DISCUSSION

In this analysis of nearly 15,000 middle-aged adults from four communities in the United States, we found that participants who self-rated their health as “poor” compared to others their age experienced higher rates of hospitalization and death, even after adjusting for demographic factors and medical history. The risk increased with each level of self-rated health below “excellent,” including even those who reported “good” health at baseline. This association was strengthened after accounting for the correlation between hospitalization and death, suggesting that modeling hospitalization and death separately underestimates the association between poor self-rated health and increased hospitalization.

Several theories exist for why poor self-rated health is independently associated with poor outcomes.^22,23^ One explanation is that self-rated health captures health information that is not measured or fully accounted for by traditional risk factors.^2^ For example, behavioral factors such as higher quality diet and greater physical activity improve both objective and subjective health, but measurement is often incomplete or imprecise. There may also be specific deleterious conditions or behaviors undisclosed by the participant, which may be captured in more non-specific measures of health. An individual’s dynamic projection of their future health, based on their own health trajectory or in comparison to others, can also influence the response.^22,24^ Another explanation is that self-rated health reflects subconscious bodily sensations that provide a direct sense of health unavailable to external observation.^2^ Lastly, self-rated health may potentially be a self-fulfilling prophesy, reinforcing beneficial behaviors in those with better self-rated health and harmful behaviors in those without.^25^

Our study provides evidence for the first explanation, but as designed, could not address the others. The unadjusted model estimated a substantial difference in event rates between the self-rated health categories which was attenuated after adjusting for medical conditions. This recapitulates findings from prior studies that suggest self-rated health is an independent risk factor for health outcomes.^8,10,11,23,26–30^ However, as demonstrated in a study that incorporated number of diseases, physical functioning, and 150 biomarkers, simply accounting for more measures of objective health does not appear to eliminate the prognostic power of self-rated health.^31^ If self-rated health and objective biomarkers indeed capture wholly different domains of health, using both in conjunction can provide more information than each individually.^32^

Previous studies of self-rated health in the ARIC study demonstrated that self-rated health declines after a diagnosis of a major disease such as myocardial infarction, stroke, heart failure, or cancer.^33,34^ Our analysis here focused on self-rated health in midlife as an exposure and tracked the accumulating morbidity and mortality over time as measured by rates of hospitalization and death. Together, these analyses provide support for the bi-directional nature of self-rated health and illness. Individuals with poor self-rated health tend to accumulate more disease, and individuals diagnosed with major diseases tend to have worsening trajectories of self-rated health.^34^

Our study is novel in that it demonstrates the longitudinal association of self-rated health with hospitalizations over three decades of follow-up time, in a large and diverse cohort of middle-aged adults. To our knowledge, no other study of recurrent hospitalizations and self-rated health has had nearly this length of outcome ascertainment. Additionally, the joint survival model provided flexibility over traditional single event or recurrent event analysis and addressed the conservative bias incurred by non-independent censoring. Because our analysis is computationally reproducible and was conducted with open-source software, future investigators can readily apply these methods to study cause-specific hospitalizations in ARIC and other populations.

Several limitations are present, which involve the measurement of self-rated health and construct validity of hospitalization as a proxy for morbidity. The particular wording of the self-rated health question in ARIC included a phrase that prompted individuals to compare their health to other people their age. This qualifier could have diminished the age-related changes in self-rated health and would complicate the direct comparison of these findings to other studies using a different wording. This study did not use information from repeated measurements of self-rated health, but more complex models using time-dependent covariates or marginal structural models could incorporate the repeated measurements to better derive causal effect estimates.^35^ Likewise, interventions that directly or indirectly improve self-rated health should be studied to examine whether improvements could decrease risk of future hospitalization or death.

In this study, each hospitalization was considered a single event with equal weight. However, hospital admissions are heterogeneous events with potentially widely differing determinants and effects with respect to self-rated health. Hospitalizations following elective or cosmetic procedures, for example, would be indicators of well-being, rather than illness. Further studies could focus on disease-specific hospitalizations and identify self-rated health trajectories or participant-level characteristics specific to a single disease process.

Our study has clinical implications because it reinforces the prognostic power of self-rated health beyond traditional risk factors, and extends our knowledge to the outcome of all-cause hospitalization. The major finding is that self-rated health status informs an individual’s future risk of hospitalization beyond factors that are conventionally observed. Clinicians can use this simple and convenient measure for individual patients to provide more accurate and personalized risk assessments. Additionally, self-rated health assessments using standardized workflows may be incorporated into routine care to predict future hospitalization rates for clinics, hospitals, and communities.^36^ Improvements in factors that influence how people self-rate their health could also improve clinical outcomes such as hospitalization risk. Thus, our study provides a rationale for future inquiry into routine assessment of self-rated health or even targeted interventions that may improve self-rated health and its determinants.

## CONCLUSION

An unfavorable response from a single assessment of self-rated health taken in middle age was strongly and independently associated with increased risks of both hospitalization and death. This simple measure can identify individuals who are at a higher risk of adverse health outcomes, beyond what is typically captured from objective measurements of health.

### Supplementary Information

Below is the link to the electronic supplementary material.

Supplementary file1 (DOCX 22.1 KB)

## Data Availability

Atherosclerosis Risk in Communities (ARIC) data is available upon request from the ARIC Data Coordinating Center (https://aric.cscc.unc.edu/aric9/researchers/Obtain_Submit_Data). The statistical code used for this analysis is available at 10.17605/OSF.IO/BEQXZ.
