# Supplementary material for: Self-Rated Health in Middle Age and Risk of Hospitalizations and Death: Recurrent Event Analysis of the ARIC Study
Source: J Gen Intern Med. 2024 Apr 10;39(10):1850–7. doi: 10.1007/s11606-024-08748-0 (PMC11282046; doi:10.1007/s11606-024-08748-0)
Supplement: Supplementary file 1 — Supplementary file1 (DOCX 22.1 KB) [file 11606_2024_8748_MOESM1_ESM.docx]

Appendix Table 1. Mean cumulative number of hospitalizations. The Nelson-Aalen estimates of the cumulative mean cumulative number of hospitalizations by self-rated health status reported at the home interview are displayed by year. This non-parametric estimate takes into account the shrinking risk set over time as individuals die or become censored, but does not take into account the potential violation of the independent censoring assumption that time to death is non-informative with respect to number of hospitalizations.

| **Year** | **Excellent** | **Good** | **Fair** | **Poor** |
| --- | --- | --- | --- | --- |
| **1987** | 0.0 (0.0-0.0) | 0.0 (0.0-0.0) | 0.0 (0.0-0.0) | 0.0 (0.0-0.0) |
| **1988** | 0.1 (0.0-0.1) | 0.1 (0.0-0.1) | 0.1 (0.1-0.2) | 0.1 (0.0-0.3) |
| **1989** | 0.1 (0.1-0.1) | 0.1 (0.1-0.2) | 0.3 (0.2-0.3) | 0.5 (0.3-0.6) |
| **1990** | 0.2 (0.1-0.2) | 0.2 (0.2-0.2) | 0.5 (0.4-0.5) | 0.8 (0.6-1.0) |
| **1991** | 0.2 (0.2-0.3) | 0.3 (0.3-0.3) | 0.7 (0.6-0.7) | 1.2 (1.0-1.4) |
| **1992** | 0.3 (0.3-0.3) | 0.4 (0.4-0.5) | 0.8 (0.8-0.9) | 1.5 (1.3-1.8) |
| **1993** | 0.4 (0.3-0.4) | 0.5 (0.5-0.6) | 1.0 (1.0-1.1) | 1.9 (1.6-2.2) |
| **1994** | 0.4 (0.4-0.5) | 0.6 (0.6-0.7) | 1.3 (1.2-1.4) | 2.3 (2.0-2.7) |
| **1995** | 0.5 (0.5-0.6) | 0.7 (0.7-0.8) | 1.5 (1.4-1.6) | 2.8 (2.4-3.1) |
| **1996** | 0.6 (0.6-0.7) | 0.9 (0.8-0.9) | 1.7 (1.6-1.8) | 3.2 (2.8-3.7) |
| **1997** | 0.7 (0.6-0.7) | 1.0 (1.0-1.1) | 2.0 (1.8-2.1) | 3.6 (3.2-4.1) |
| **1998** | 0.8 (0.7-0.8) | 1.1 (1.1-1.2) | 2.2 (2.1-2.4) | 4.1 (3.7-4.6) |
| **1999** | 0.9 (0.8-0.9) | 1.3 (1.2-1.3) | 2.5 (2.4-2.7) | 4.6 (4.1-5.2) |
| **2000** | 1.0 (0.9-1.1) | 1.5 (1.4-1.5) | 2.8 (2.6-3.0) | 5.1 (4.5-5.7) |
| **2001** | 1.1 (1.1-1.2) | 1.6 (1.6-1.7) | 3.1 (2.9-3.3) | 5.5 (4.9-6.1) |
| **2002** | 1.2 (1.2-1.3) | 1.8 (1.7-1.9) | 3.4 (3.2-3.6) | 6.0 (5.3-6.7) |
| **2003** | 1.4 (1.3-1.4) | 2.0 (1.9-2.1) | 3.7 (3.5-3.9) | 6.4 (5.7-7.1) |
| **2004** | 1.5 (1.4-1.6) | 2.2 (2.1-2.3) | 4.1 (3.9-4.3) | 7.0 (6.2-7.7) |
| **2005** | 1.6 (1.6-1.7) | 2.4 (2.3-2.5) | 4.4 (4.2-4.6) | 7.5 (6.7-8.3) |
| **2006** | 1.8 (1.7-1.8) | 2.6 (2.5-2.7) | 4.7 (4.5-5.0) | 8.1 (7.3-9.0) |
| **2007** | 1.9 (1.8-2.0) | 2.8 (2.7-2.9) | 5.1 (4.8-5.3) | 8.8 (7.9-9.7) |
| **2008** | 2.1 (2.0-2.2) | 3.0 (2.9-3.1) | 5.4 (5.1-5.7) | 9.4 (8.4-10.3) |
| **2009** | 2.2 (2.1-2.3) | 3.3 (3.2-3.4) | 5.8 (5.5-6.0) | 10.0 (8.9-11.0) |
| **2010** | 2.4 (2.3-2.5) | 3.5 (3.4-3.6) | 6.1 (5.8-6.4) | 10.6 (9.5-11.8) |
| **2011** | 2.6 (2.5-2.7) | 3.7 (3.6-3.9) | 6.4 (6.1-6.8) | 11.3 (10.1-12.4) |
| **2012** | 2.8 (2.7-2.9) | 4.0 (3.9-4.1) | 6.8 (6.5-7.1) | 11.9 (10.6-13.1) |
| **2013** | 3.0 (2.9-3.1) | 4.3 (4.1-4.4) | 7.2 (6.9-7.5) | 12.5 (11.2-13.9) |
| **2014** | 3.2 (3.1-3.3) | 4.6 (4.4-4.7) | 7.6 (7.3-8.0) | 13.3 (11.9-14.6) |
| **2015** | 3.4 (3.3-3.5) | 4.9 (4.7-5.0) | 8.0 (7.7-8.4) | 13.7 (12.3-15.1) |
| **2016** | 3.6 (3.5-3.8) | 5.1 (5.0-5.3) | 8.5 (8.1-8.8) | 14.2 (12.8-15.7) |
| **2017** | 3.9 (3.7-4.0) | 5.4 (5.3-5.6) | 8.9 (8.5-9.3) | 14.7 (13.2-16.2) |
| **2018** | 4.2 (4.0-4.3) | 5.8 (5.6-6.0) | 9.4 (9.0-9.8) | 15.9 (14.2-17.5) |
| **2019** | 4.4 (4.3-4.6) | 6.1 (6.0-6.3) | 9.8 (9.4-10.3) | 16.9 (15.0-18.7) |

Appendix table 2. Joint frailty model for hospitalization rate and mortality hazard. The coefficient estimates for the final joint frailty model (Model 3) with demographic and clinical factors added as linear adjustment terms are displayed with the 95% confidence intervals within parentheses. This model takes into account the association between risk of hospitalization and hazard of death.

|  | **Hospitalizations** | | **Deaths** | |
| --- | --- | --- | --- | --- |
| **Home Interview Self-Rated Health** | **Rate Ratio (95% CI)** | **p-value** | **Hazard Ratio(95% CI)** | **p-value** |
| **Excellent** | Reference | — | Reference | — |
| **Good** | 1.22 (1.07 to 1.40) | 0.003 | 1.30 (1.12 to 1.51) | 0.001 |
| **Fair** | 2.01 (1.63 to 2.47) | <0.001 | 2.15 (1.71 to 2.69) | <0.001 |
| **Poor** | 3.13 (2.39 to 4.09) | <0.001 | 3.40 (2.54 to 4.56) | <0.001 |
| **Forsyth County (Black)** | Reference | — | Reference | — |
| **Forsyth County (White)** | 0.79 (0.55 to 1.14) | 0.202 | 0.77 (0.51 to 1.16) | 0.209 |
| **Jackson, Mississippi (Black)** | 0.85 (0.59 to 1.23) | 0.399 | 0.99 (0.65 to 1.49) | 0.947 |
| **Minneapolis, Minnesota (White)** | 0.83 (0.57 to 1.21) | 0.330 | 0.77 (0.52 to 1.16) | 0.216 |
| **Washington County (White)** | 1.08 (0.73 to 1.59) | 0.693 | 1.02 (0.67 to 1.54) | 0.939 |
| **Female** | Reference | — | Reference | — |
| **Male** | 1.13 (1.02 to 1.26) | 0.018 | 1.52 (1.36 to 1.71) | <0.001 |
| **Age (1 year increase)** | 1.06 (1.05 to 1.07) | <0.001 | 1.13 (1.12 to 1.15) | <0.001 |
| **Normal weight** | Reference | — | Reference | — |
| **Underweight** | 1.27 (0.82 to 1.95) | 0.283 | 1.49 (0.91 to 2.43) | 0.115 |
| **Overweight** | 1.02 (0.88 to 1.19) | 0.789 | 0.90 (0.76 to 1.06) | 0.187 |
| **Obese** | 1.07 (0.93 to 1.23) | 0.371 | 1.00 (0.85 to 1.18) | 0.987 |
| **Never smoker** | Reference | — | Reference | — |
| **Current smoker** | 1.79 (1.55 to 2.07) | <0.001 | 2.99 (2.54 to 3.52) | <0.001 |
| **Former smoker** | 1.29 (1.12 to 1.48) | <0.001 | 1.40 (1.20 to 1.63) | <0.001 |
| **Cancer** | 1.46 (1.15 to 1.84) | 0.002 | 1.64 (1.26 to 2.14) | <0.001 |
| **COPD/Emphysema** | 1.11 (0.93 to 1.32) | 0.255 | 1.09 (0.90 to 1.34) | 0.373 |
| **Coronary heart disease** | 1.43 (0.95 to 2.16) | 0.089 | 1.18 (0.71 to 1.94) | 0.523 |
| **Myocardial infarction** | 0.99 (0.65 to 1.50) | 0.961 | 1.33 (0.80 to 2.21) | 0.271 |
| **Heart failure** | 1.54 (1.27 to 1.88) | <0.001 | 1.54 (1.23 to 1.92) | <0.001 |
| **Hypertension** | 1.33 (1.17 to 1.51) | <0.001 | 1.42 (1.23 to 1.63) | <0.001 |
| **Diabetes** | 1.65 (1.41 to 1.93) | <0.001 | 2.04 (1.72 to 2.43) | <0.001 |

Appendix table 3. Cox proportional hazards model for mortality. The coefficient estimates for the Cox proportional hazards model that estimated the relative hazards of death ignoring hospitalization information are displayed with the 95% confidence intervals within parentheses.

|  | **Death** | |
| --- | --- | --- |
| **Covariate** | **Hazard Ratio (95% CI)** | **p-value** |
| **Excellent** | Reference | — |
| **Good** | 1.25 (1.18 to 1.33) | <0.001 |
| **Fair** | 1.64 (1.52 to 1.76) | <0.001 |
| **Poor** | 2.35 (2.09 to 2.65) | <0.001 |
| **Forsyth County (Black)** | Reference | — |
| **Forsyth County (White)** | 1.00 (0.87 to 1.15) | 0.986 |
| **Jackson, Mississippi (Black)** | 1.16 (1.01 to 1.34) | 0.034 |
| **Minneapolis, Minnesota (White)** | 1.00 (0.87 to 1.16) | 0.950 |
| **Washington County (White)** | 1.11 (0.96 to 1.27) | 0.156 |
| **Female** | Reference | — |
| **Male** | 1.48 (1.41 to 1.55) | <0.001 |
| **Age (1 year increase)** | 1.11 (1.11 to 1.12) | <0.001 |
| **Normal weight** | Reference | — |
| **Underweight** | 1.49 (1.20 to 1.85) | <0.001 |
| **Overweight** | 1.00 (0.94 to 1.06) | 0.960 |
| **Obese** | 1.23 (1.16 to 1.31) | <0.001 |
| **Never smoker** | Reference | — |
| **Current smoker** | 2.41 (2.28 to 2.56) | <0.001 |
| **Former smoker** | 1.20 (1.13 to 1.27) | <0.001 |
| **Cancer** | 1.21 (1.11 to 1.33) | <0.001 |
| **COPD/Emphysema** | 1.31 (1.20 to 1.43) | <0.001 |
| **Coronary heart disease** | 1.32 (1.06 to 1.65) | 0.013 |
| **Myocardial infarction** | 1.40 (1.11 to 1.77) | 0.005 |
| **Heart failure** | 1.14 (1.04 to 1.26) | 0.007 |
| **Hypertension** | 1.29 (1.22 to 1.36) | <0.001 |
| **Diabetes** | 1.75 (1.64 to 1.86) | <0.001 |

Appendix table 4. Andersen-Gill proportional intensity model for hospitalization rate. The coefficient estimates for the Andersen-Gill proportional intensity model that estimated the relative intensity of recurrent hospitalization events, treating death as an independent censoring mechanism, are displayed with the 95% confidence intervals within parentheses.

|  | **Hospitalizations** | |
| --- | --- | --- |
| **Covariate** | **Intensity Ratio (95% CI)** | **p-value** |
| **Excellent** | Reference |  |
| **Good** | 1.21 (1.18 to 1.23) | <0.001 |
| **Fair** | 1.62 (1.58 to 1.66) | <0.001 |
| **Poor** | 2.35 (2.26 to 2.45) | <0.001 |
| **Forsyth County (Black)** | Reference |  |
| **Forsyth County (White)** | 1.00 (0.96 to 1.05) | 0.973 |
| **Jackson, Mississippi (Black)** | 1.00 (0.95 to 1.05) | 0.965 |
| **Minneapolis, Minnesota (White)** | 1.04 (0.99 to 1.09) | 0.589 |
| **Washington County (White)** | 1.14 (1.09 to 1.20) | 0.063 |
| **Female** | Reference |  |
| **Male** | 1.11 (1.09 to 1.13) | <0.001 |
| **Age (1 year increase)** | 1.04 (1.04 to 1.04) | <0.001 |
| **Normal weight** | Reference |  |
| **Underweight** | 1.23 (1.13 to 1.34) | 0.027 |
| **Overweight** | 1.16 (1.13 to 1.18) | <0.001 |
| **Obese** | 1.37 (1.34 to 1.40) | <0.001 |
| **Never smoker** | Reference |  |
| **Current smoker** | 1.55 (1.52 to 1.58) | <0.001 |
| **Former smoker** | 1.13 (1.11 to 1.15) | <0.001 |
| **Cancer** | 1.10 (1.06 to 1.14) | 0.016 |
| **COPD/Emphysema** | 1.33 (1.29 to 1.37) | <0.001 |
| **Coronary heart disease** | 1.59 (1.48 to 1.71) | <0.001 |
| **Myocardial infarction** | 1.08 (1.00 to 1.17) | 0.333 |
| **Heart failure** | 1.13 (1.09 to 1.17) | 0.005 |
| **Hypertension** | 1.23 (1.21 to 1.26) | <0.001 |
| **Diabetes** | 1.55 (1.51 to 1.58) | <0.001 |
